# Advanced glycation end products induce endothelial hyperpermeability via β‐catenin phosphorylation and subsequent up‐regulation of ADAM10

**DOI:** 10.1111/jcmm.16659

**Published:** 2021-07-05

**Authors:** Jie Weng, Zhenfeng Chen, Jieyu Li, Qi He, Deshu Chen, Lin Yang, Haiying Su, Junlin Huang, Shengxiang Yu, Qiaobing Huang, Qiulin Xu, Xiaohua Guo

**Affiliations:** ^1^ Department of Pulmonary and Critical Care Medicine Zhujiang Hospital Southern Medical University Guangzhou China; ^2^ Department of Pathophysiology Guangdong Provincial Key Laboratory of Shock and Microcirculation School of Basic Medical Sciences Southern Medical University Guangzhou China; ^3^ Department of Emergency and Critical Medicine Guangdong Provincial People’s Hospital Guangdong Academy of Medical Science Guangzhou China; ^4^ Guangzhou Special Service Sanatorium Center of the Rocket Force Guangzhou China

**Keywords:** advanced glycation end products, β‐catenin, ADAM10, adherens junctions, hyperpermeability

## Abstract

Endothelial hyperpermeability is the initial event in the development of diabetic microvascular complications, and advanced glycation end products (AGEs) are suggested to cause much of the endothelial hyperpermeability associated with diabetes mellitus, but the molecular mechanism remains to be characterized. β‐catenin reportedly plays dual functions in maintaining normal endothelial permeability by serving both as an adhesive component and a signal transduction component. Here, we found that AGEs induced the phosphorylation of β‐catenin at residues Y654 and Y142 and the endothelial hyperpermeability was reversed when the two residues were blocked. In mechanism, phosphorylation of Y654 was blocked by Src inactivation, whereas phosphorylation of Y142 was reduced by a focal adhesion kinase inhibitor. β‐catenin Y654 phosphorylation induced by AGEs facilitated the dissociation of vascular endothelial (VE)‐cadherin/β‐catenin and the impairment of adherens junctions (AJs), whereas β‐catenin Y142 phosphorylation favoured the dissociation of β‐catenin and α‐catenin. Further investigation revealed that β‐catenin Y142 phosphorylation was required for AGEs‐mediated β‐catenin nuclear translocation, and this nuclear‐located β‐catenin subsequently activated the TCF/LEF pathway. This pathway promotes the transcription of the Wnt target, ADAM10 (a disintegrin and metalloprotease 10), which mediates VE‐cadherin shedding and leads to further impairment of AJs. In summary, our study showed the role of β‐catenin Y654 and Y142 phosphorylation in AGEs‐mediated endothelial hyperpermeability through VE‐cadherin/β‐catenin/α‐catenin dissociation and up‐regulation of ADAM10, thereby advancing our understanding of the underlying mechanisms of AGEs‐induced microvascular hyperpermeability.

## 1 INTRODUCTION

Diabetes mellitus is a highly prevalent chronic disease that adversely affects the diffusive microvasculature in the body.^1^ The microvascular injury resulting from sustained hyperglycaemia causes multiple organ dysfunction and, hence, serves as one of the major factors leading to diabetic morbidity and mortality. Researchers have shown a strong link between diabetic microvascular complications and advanced glycation end products (AGEs).^2^, ^3^ The AGEs are proteins or lipids that become glycated upon exposure to sugars. These sugars are present in excess in a hyperglycaemic environment. Generally, the contribution of AGEs towards diabetic complications is both through their direct interactions with proteins, which adversely affects protein structure and/or function, or through engagement with the receptor for advanced glycation end products (RAGE), which perturbs various cellular properties.^2^, ^3^ For example, the activation of RAGE compromises microvascular barrier function, leading to endothelial hyperpermeability.^4^, ^5^ More specifically, the combination of AGEs and RAGE has been reported to trigger the disruption of endothelial adherens junctions (AJs) in vitro.^6^

Vascular endothelial (VE)‐cadherin acts as a cornerstone of AJs and regulates the calcium‐dependent cell‐cell adhesion that holds neighbouring cells together and regulates the opening and closing of the endothelial cells. Disruption of AJs has been suggested to be the leading cause of endothelial hyperpermeability in response to pathological stimuli such as thrombin, TNF‐α and lipopolysaccharides.^7^, ^8^ Numerous studies aiming to uncover the mechanisms involved in the dynamic regulation of AJs suggest that the phosphorylation of AJ components contributes towards junction weakening and the opening of the endothelial barrier.^9^, ^10^, ^11^ Indeed, our previous studies presented enhanced phosphorylation of VE‐cadherin in AGEs‐induced endothelial hyperpermeability.^12^ Notably, another study reported that the direct phosphorylation of VE‐cadherin by Src appears insufficient to mediate endothelial barrier dysfunction.^13^ Thus, additional factors may drive the disruption of AJs in association with AGEs‐induced endothelial hyperpermeability.

β‐catenin acts as a communication bridge between the intracellular segment of cadherin and the cytoskeleton.^14^, ^15^ Physiologically, β‐catenin is recruited to the cytosolic tail of VE‐cadherin to protect these cadherins from proteasomal degradation.^16^, ^17^ Veelen et al provided in vivo evidence confirming that tyrosine phosphorylation of β‐catenin at Y654 reduces its affinity towards E‐cadherin.^18^ Additionally, studies have shown that introducing Y654‐mutant β‐catenin into L‐cells rescued the disassembly of AJs in response to histamine.^19^ Moreover, β‐catenin phosphorylation has been reported to regulate N‐cadherin‐mediated cell‐cell adhesion.^17^ However, the effect of the AJ disruption caused by phosphorylation of β‐catenin at Y654 on permeability in endothelial cells remains to be studied in the context of AGEs exposure. Besides the vital role of β‐catenin Y654 phosphorylation, Y142 phosphorylation also plays an important part in regulating β‐catenin function—the Tyr142‐phosphorylated β‐catenin stimulates vascular cell‐adhesion molecule‐1 expression to increase endothelial cell (EC)‐monocytic adhesion; mutation of β‐catenin at Y142 disrupts the dissociation of the MET receptor tyrosine kinase/β‐catenin complex.^20^, ^21^ A single amino acid mutation at Y142 to simulate the phosphorylation state (termed a phospho‐mimic) significantly reduces its binding with α‐catenin.^22^ The β‐catenin/α‐catenin complex is known to stabilize the β‐catenin in AJs,^23^ whereas disruption of this complex contributes towards the α‐catenin homodimer associating with F‐actin instead.^24^

In addition to its adhesive function, β‐catenin also serves as a signal transduction component. Clevers *et al* demonstrated that β‐catenin that dissociates in the cytoplasm is transferred into the nucleus with the help of B‐cell CLL/lymphoma 9 (BCL9), where it combines with T‐cell factor/lymphoid enhancer factor (TCF/LEF).^25^ This initiates the transcription of Wnt‐related genes encoding cMyc and Cyclin D1. A disintegrin and metalloprotease 10 (ADAM10) acts as an ectodomain sheddase and is a target gene of the Wnt/β‐catenin pathway.^26^ Further works have identified ADAM10 as a regulator of vascular permeability through its direct proteolytic cleavage of VE‐cadherin, which releases a soluble fragment and generates a carboxyl‐terminal membrane‐bound stub.^7^, ^27^

Src family kinases (SFKs) are the family of non‐receptor tyrosine kinase. As signalling enzymes, they consist of nine structurally related proteins, Src, Blk, Fyn, Yes, Lyn, Lck, Hck, Fgr and Yrk. Src is one of the most widely studied members in SFKs.^28^, ^29^ SFKs play an important role in proliferation, apoptosis, migration, angiogenesis, cell‐cell adhesion and communication. SFKs regulate normal and pathological processes in vascular biology, including proliferation and permeability of endothelial cells. And they are known to play an important role in promoting endothelial permeability through mechanisms of paracellular and transcellular transport. For example, SFKs aﬀect vascular permeability through regulating focal adhesion complexes, containing integrins, focal adhesion kinase (FAK) and multiple adaptor proteins.

Based on these previous observations, we suggested that the phosphorylation of β‐catenin at Y654 and Y142 affects the regulation of AJs and F‐actin remodelling, respectively, whereas the latter further activates the Wnt/β‐catenin pathway and promotes ADAM10 transcription. Thus, in this study, we investigated the role of β‐catenin phosphorylation in the pathology of AGEs‐induced endothelial hyperpermeability.

## 2 MATERIALS AND METHODS

### 2.1 Transfection of siRNA and plasmids

siRNA transfection was carried out according to protocol provided by GenePharma (Shanghai, China). Briefly, HUVECs were cultured in 6‐well plates with 50%~ 70% confluences and then transfected with 20 nmol/L siRNA through siRNA‐Mate^TM^. 48 hours after transfection, the cells were treated with or without AGEs. Then, total protein was prepared and subjected to Western blot. The sequences targeted to Src were TGTTCGGAGGCTTCAACTCCT, and ADAM10 was CAGCAGAGAGAUAUAUUAATT whereas Control siRNA were AATTCTCCGAACGTGTCACGT, which were synthesized by GenePharma (Shanghai, China).

The transfection of plasmids was performed according to Lipofectamine^TM^ kit. Briefly, cells were cultured to 90%‐100% confluences and then transfected with DNA complexes for 48‐72 hours, followed by treatments with or without AGEs. Total proteins were extracted and were detected to verify the transduction efficiency of siRNA or plasmid vectors, with the results shown in Figure S4A and Figure S5A,D.

The cells lysates were collected and subjected to co‐immunoprecipitation and immunofluorescence to detect the AJs colocation, Western blot to detect ADAM10 protein level and VE‐cadherin shedding, Gene reporter assays to detect TCF/LEF promoter activity.

### 2.2 Preparation of AGEs

AGEs‐modified bovine serum albumin (AGEs‐BSA) was prepared based on the protocol of Schmidt AM et al^30^ with slight modification. Briefly, BSA (50 mg/mL, pH 7.4) was incubated in PBS with D‐glucose (100 mmol/L) at 37°C for 8 weeks. Control albumin was incubated without glucose. Both solutions were extensively concentrated and purified at the end of the incubation period. AGEs‐specific fluorescence (excitation, 370 nm; emission, 440 nm)^31^ was determined using ratio spectrofluorometry. AGEs contained 75.20 U/mg proteins, whereas native albumin contained less than 0.9 U/mg proteins of AGEs. The endotoxin content was detected using TCL kit and was found to be <0.5 EU/mL in both solutions.

### 2.3 Quantitative real‐time PCR

Total RNA was isolated from cells with TRIzol reagent (#D1105, GBCBIO Technologies, China). The RNA concentration was determined by using a BioDrop spectrophotometer (Biochrom Ltd, Cambridge, UK). Complementary DNA was reverse transcribed with HiScript^®^ III RT SuperMix (#R323, Vazyme) according to the manufacturer's protocol. Real‐time PCR was performed using SYBR® qPCR Master Mix(#Q331, Vazyme) with specific primers(Table 1) in a 7500 Real‐Time PCR System (Applied Biosystems, Foster City, CA). Beta‐actin was used as the internal control. The 2^−ΔΔCt^ method was employed to evaluate mRNA expression.

**TABLE 1:** Sequences of primers for qRT‐PCR

Gene name	Forward primer	Reverse primer
*ADAM10*	5′‐ATGGGAGGTCAGTATGGGAATC‐3′	5′‐ACTGCTCTTTTGGCACGCT‐3′
*MMP2*	5′‐AGCGAGTGGATGCCGCCTTTAA‐3′	5′‐CATTCCAGGCATCTGCGATGAG‐3′
*MMP7*	5′‐TCGGAGGAGATGCTCACTTCGA‐3′	5′‐GGATCAGAGGAATGTCCCATACC‐3′
*MMP9*	5′‐GCCACTACTGTGCCTTTGAGTC‐3′	5′‐CCCTCAGAGAATCGCCAGTACT‐3′
*CCND1*	5′‐TCTACACCGACAACTCCATCCG‐3′	5′‐TCTGGCATTTTGGAGAGGAAGTG‐3′
*VEGFA*	5′‐ATCTTCAAGCCATCCTGTGTG‐3′	5′‐GAGGTTTGATCCGCATAATCTG‐3′
*Beta‐Actin*	5′‐TCCACCTTCCAGCAGATGTG‐3′	5′‐GCATTTGCGGTGGACGAT‐3′

### 2.4 Western blotting

Cells were lysed using Lysis buffer containing phosphatase and protease inhibitors. After extraction, total proteins were first subjected to 10% SDS‐PAGE separation and then transferred to polyvinylidene difluoride membranes and subsequently blocked with 5% BSA dissolved in 1% TBST, incubated with specific primary antibodies of 1:1000 dilution overnight at 4°C. After three times washing, the membranes were incubated with respective secondary antibodies at room temperature for 1 hour, and signal was detected by chemiluminescence. Finally, analysis of band density was carried out by Tiangen imaging station and quantified with ImageJ.

### 2.5 Co‐Immunoprecipitation

HUVEC lysate was harvested using 200 μL Cell Lysis Buffer for IP (Beyotime biotechnology, Cat No. P0013) with protease and phosphatase inhibitors, and then immunoprecipitation was performed according to protocol provided by Millipore. In brief, 200 μL total proteins were firstly incubated with 2 μL β‐catenin antibody overnight at 4°C and subsequently mixed with 40 μL Pure Proteome^TM^ Protein A/G Mix Magnetic Beads at room temperature for 30 min. Using magnetic stand to capture the beads, then remove the supernatant sample and disengage the magnet and gently wash the beads with binding/wash buffer. After the last wash, disengage the magnet and add 1×loading buffer to denature the elution.

### 2.6 Endothelial cells permeability assay

Measurement of transendothelial electrical resistance (TER) and FITC‐Dextran transendothelial flux was performed to evaluate endothelial permeability. As us published previously,^12^ for TER measurement, HUVECs were incubated on the upper chamber of 0.4 μm‐pore‐size‐transwell (US，Coming Costar) to 100% confluence and stimulated with 100 μg/mL AGEs for corresponding time, then EVOM^2^ (World Precision Instruments, USA) was used to measure monolayer permeability. To test transendothelial flux of FITC‐Dextran, cells were seeded on the upper chamber and incubated with FITC‐labelled dextran (1 mg/mL) for 45 min, then immediately collected 200 μL supernatant in the upper and bottom chamber, respectively, and determined the concentration of FITC‐dextran using HTS 7000 microplate reader. The permeability coefficient of dextran was calculated as follows: Pd = [A]/t × 1/A × V/[L], with [A] indicates the concentration of bottom chamber, [L] represents the upper ones, t represents time in seconds, A refers to the membrane area (in cm^2^), and V indicates the volume of the bottom chamber.

### 2.7 Immunofluorescent staining

HUVECs were seeded on the microporous petri dish and cultured to 80%~ 90% confluences following with 8h exposure of 100 μg/mL AGEs after β‐catenin Y142F plasmid transduction. After washed briefly with phosphate‐buffered saline (PBS), cells were firstly fixed with 4% formaldehyde for 10 minutes and then permeabilized in 0.5% Triton X‐100 for 10 minutes at room temperature. Afterwards, cells were blocked with 5% BSA and subjected to incubation of primary antibody overnight at 4°C. Next, the samples were incubated with appropriate Alexa‐Fluor‐coupled secondary antibodies. Finally, DAPI (1:1000) was administrated to label the nucleus, and Zeiss LSM780 laser confocal scanning microscope (Zeiss, Germany) was used to detect the immunofluorescent signals.

### 2.8 In vivo assay of microvascular permeability

Wild‐type C57BL/6 mice (aged 6‐8 weeks) were acquired from the laboratory centre of Southern Medical University. The protocol was approved by the Animal Care Committee of the Southern Medical University of China and was in accordance with the National Institutes of Health guidelines for ethical animal treatment. According to the protocol of Moore et al,^32^ mice were administered intraperitoneally with PBS or PBS‐diluted AGEs (10 mg/kg) for 7 consecutive days. Indicated adenoviruses were given i.v. 2 days before PBS or AGEs injection. Then, mice were anaesthetised with intraperitoneal pentobarbital sodium (50 mg/kg) followed by cannulation of jugular veins and intravenously injection of FITC‐dextran at 15 mg/kg with continuous infusion at 15 mg/kg/min. Sequentially, mice were placed on a Plexiglas platform mounted to an intravital upright microscope and a midline laparotomy was performed; then, the mesenteric venules were selected for FITC‐dextran transvascular flux measurement. Mesenteric transvascular flux was calculated with the following equation: Δ*I* = 1 − (*I_i_* − *I_o_*)/*I_i_*. ΔI indicates changes in light intensity. I*_i_* refers to the light intensity inside the vessel, and *I_o_* means the light intensity outside the vessel. The excitation wave used to detect ﬂuorescence was 488 nm, whereas the emission wave was 525 nm.

### 2.9 Evans blue assays

C57 mice were intravenously injected with 200 μL 0.5%(w/v) Evans blue(EB) in saline through its lateral tail vein. After 2 hours, the mice were performed thoracotomy under pentobarbital sodium (50 mg/kg) anaesthesia and the heart perfused with saline to remove blood. Lungs subsequently were harvested, photographed, weighed and each placed in 2 mL tubes with 1 mL formamide and the tubes transferred to a 50°C water bath for 48h. Extravasation of EB into the interstitial tissue was quantified at 620nm wave length. Calculation formula: EB amount per gram of lung tissue (μg/g) = EB concentration (μg/mL) *1(mL)/ lung weight (g).

### 2.10 Gene reporter assays

Dual‐Luciferase Assay (Promega, WI, USA) was applied to quantify Wnt/β‐catenin signalling through TCF/LEF promoter activity in HUVECs treated with AGEs. Cells were transiently co‐transfected with TCF/LEF promoter luciferase and Renilla plasmids, at a ratio of 10:1 according to Lipofectamine^TM^ kit. Luminescence was measured by using SpectraMax M5 Multifunctional enzyme marker (Molecular Devices, CA, USA) 24 hours after transfection. Experiments were performed in triplicate and repeated twice in independent conditions. Luminescence was normalized to *Renilla* to control for transfection efficiency and expressed as fold increase compared with that of control cells.

### 2.11 Statistical analysis

All data were analysed by SPSS16.0 software and presented with mean ± SD with more than three independent experiments. One‐way ANOVA was used in statistical comparisons and LSD post hoc analysis was used to compare data among multiple groups with significant level set at *P* <.05.

## 3 RESULTS

### 3.1 AGEs induce endothelial barrier dysfunction *in* *vitro*

**FIGURE 1:**
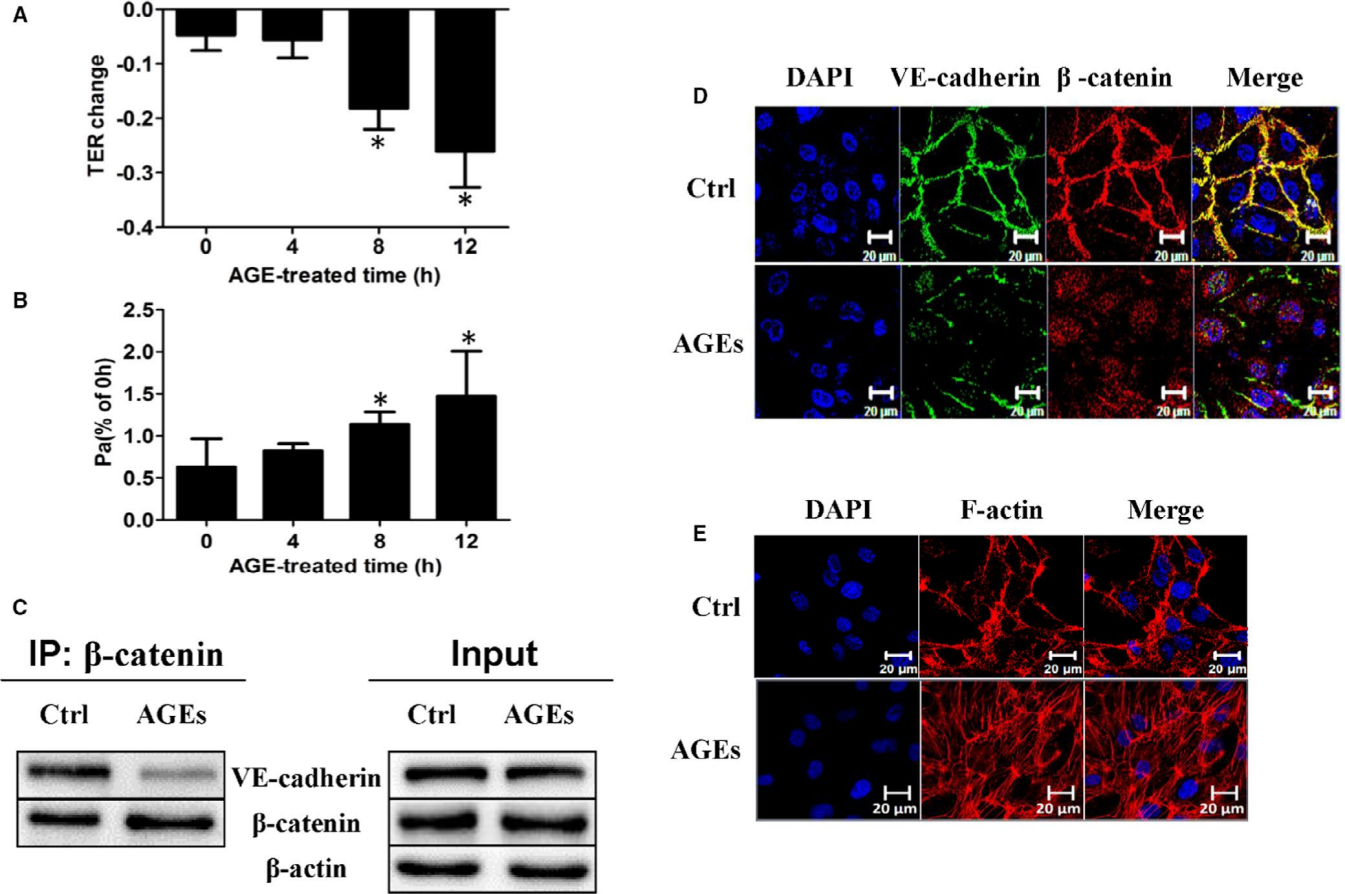
AGEs mediate endothelial barrier dysfunction in vitro. (A, B) HUVECs were stimulated by AGEs in time‐dependent manner, and TER value (n = 6) and permeability coefficient for dextran (Pa) (n = 5) were measured. Data are shown as means ± SD. *P <.05 versus 0 h. (C) HUVECs were incubated with or without AGEs for 60 min. Co‐Immunoprecipitation of VE‐cadherin and β‐catenin was performed and shown in the IP column, whereas total proteins were presented in the Input column. (D, E) HUVECs were treated with or without 100 μg/mL AGEs for 8 h, and representative confocal images of VE‐cadherin/β‐catenin and F‐actin were presented

Firstly, our results showed that AGEs time‐dependently induced endothelial monolayer hyperpermeability, as indicated by decreased TER (Figure 1A) and the increased permeability coefficient for dextran (Pa) (Figure 1B). When HUVECs were incubated with 100 μg/mL AGEs for 1 hour, VE‐cadherin/β‐catenin dissociation was detected, whereas the total protein levels of VE‐cadherin and β‐catenin remained unchanged (Figure 1C). The HUVECs were then stimulated with 100 μg/mL AGEs for 8 hours and immunofluorescence staining showed impairment of the adherence junctions (AJs) based on dispersed VE‐cadherin in the cytoplasm rather than in the cell membrane and nuclear translocation of β‐catenin (Figure 1D). We further observed that AGEs induced actin remoulding in endothelial cells, as indicated by the transfer of peripherally localized F‐actin into centralized stress fibres (Figure 1E).

### 3.2 AGEs induce β‐catenin Y654 and Y142 phosphorylation, which requires Src and FAK, respectively

**FIGURE 2:**
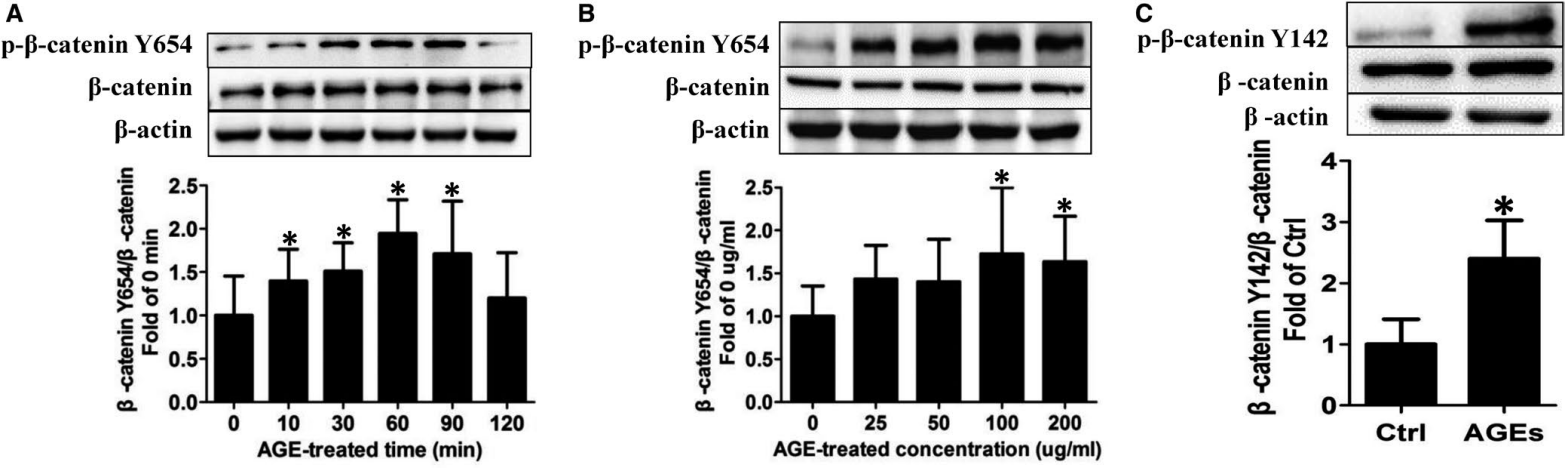
AGEs induce β‐catenin Y654 and Y142 phosphorylation. (A) HUVECs were stimulated by 100 μg/mL AGEs in time‐dependent manner. β‐catenin Y654 phosphorylation was detected by Western blotting (n = 14). Data are shown as mean ± SD. *P <.05 versus 0 min. (B) Dose‐dependent effects of AGEs on β‐catenin Y654 phosphorylation was detected (n = 10). Data are shown as mean ± SD. *P <.05 versus 0 μg/mL. (C) HUVECs were incubated with 100 μg /mL AGEs for 60 min followed by detection of β‐catenin Y142 phosphorylation (n = 4). Data are shown as mean ± SD. *P <.05 versus control

To investigate whether phosphorylation of β‐catenin at Y654 and Y142 is involved in AGE‐induced signalling events, HUVECs were incubated with 100 μg/mL AGEs, following which β‐catenin Y654 and Y142 phosphorylation were detected by Western blot. The results showed that β‐catenin Y654 phosphorylation rapidly increased with a significant elevation (*P* <.05) already observed after 10 minutes; phosphorylation then reached a peak at 60 minutes and finally returned to normal levels after 120 minutes (Figure 2A). Next, the HUVECs were stimulated with different concentrations of AGEs, and the level of β‐catenin Y654 phosphorylation was measured. The AGEs increased Y654 phosphorylation in a concentration‐dependent manner and this effect reached a peak at 100 μg/mL AGEs (Figure 2B). We next investigated whether it was involved in the observed AGEs‐induced processes. As the results showed, compared with the AGEs group, HUVECs treated with the Src specific inhibitor, PP2, before AGE stimulation (100 μg/mL for 1 hour) showed decreased β‐catenin Y654 phosphorylation (Figure S1A), implying that PP2 attenuates AGEs‐induced phosphorylation of β‐catenin at Y654. Consistent with this finding, we further observed that the genetic knock‐down of Src with siRNA abolished AGEs‐induced β‐catenin Y654 phosphorylation (Figure S1B). Thus, Src is responsible for AGEs‐induced β‐catenin Y654 phosphorylation.

Besides the AGEs‐induced phosphorylation of β‐catenin Y654 by Src, we further found that AGEs can also phosphorylate β‐catenin Y142. To determine this, HUVECs were incubated with AGEs for 1 hour and β‐catenin Y142 phosphorylation was detected by Western blot. The results showed that β‐catenin Y142 phosphorylation was significantly increased following AGEs stimulation compared with the control group (Figure 2C). At the same time, we suggested that FAK is involved in AGEs‐induced β‐catenin Y142 phosphorylation. Indeed, the introduction of PF573228, an inhibitor of FAK, blocked the effect of AGEs on β‐catenin Y142 phosphorylation (Figure S1C), suggesting that AGEs may mediate β‐catenin Y142 phosphorylation through FAK.

What's more, to explore whether FAK was the downstream target of Src in AGEs‐treated HUVECs, cells were pretreated with PP2 before the application of AGEs. We found that AGEs treatment induced a significant increase in FAK phosphorylation and this increase was inhibited by PP2 (Figure S2). Taken together, AGEs‐induced FAK activation requires Src.

### 3.3 β‐catenin Y654 phosphorylation is involved in AGEs‐mediated impairment of adherens junctions and endothelial monolayer hyperpermeability

**FIGURE 3:**
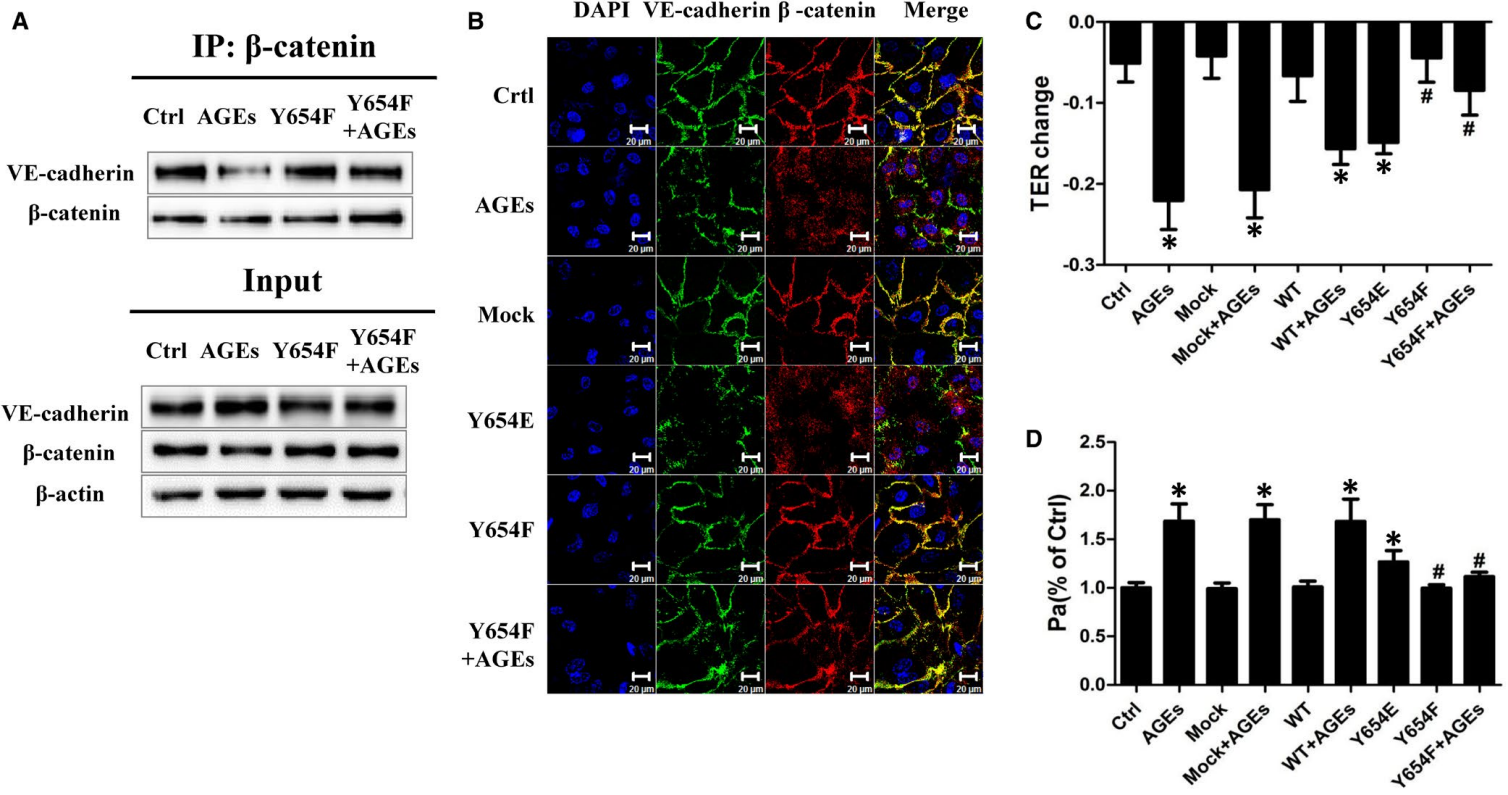
β‐catenin Y654 phosphorylation is required for AGEs‐mediated AJs disruption and endothelial hyperpermeability. A, HUVECs were pretreated with Y654F plasmid followed by AGEs exposure for 60 min. Co‐Immunoprecipitation of VE‐cadherin and β‐catenin was performed and shown in the IP column, whereas total proteins were shown in the Input column. B, HUVECs were transfected with indicated overexpression vectors followed by 8 h exposure of AGEs. Representative confocal images of the VE‐cadherin/β‐catenin were presented. C, D, HUVECs were transfected with indicated overexpression vectors followed by AGEs stimulation for 8 h. TER value and permeability coefficient for dextran (Pa) were measured (n = 5). Data are shown as mean ± SD. *P <.05 versus control, *^#^P* <.05 versus AGEs

Since our experiments showed that AGEs cause impairment of AJs and can also induce β‐catenin Y654 phosphorylation, the next step was to determine whether the phosphorylation of β‐catenin at Y654 is involved in this AGEs‐mediated disruption of the AJs. Therefore, we constructed separate vectors to overexpress either a β‐catenin Y654 phospho‐deficient mutant (Y654F) or a β‐catenin Y654 phospho‐mimic (Y654E). In contrast with the previous experiment with AGEs‐treated HUVECs that showed VE‐cadherin/β‐catenin dissociation and unchanged levels of either protein, cells pre‐transfected with the Y654F plasmid before AGEs exposure for 1 hour presented ameliorated dissociation of VE‐cadherin/β‐catenin (Figure 3A). Similarly, the immunofluorescence results showed obviously rescued AJs in cells transfected with the Y654F plasmid before AGEs stimulation for 8 hours, with normal VE‐cadherin levels and enhanced membrane localization of β‐catenin (Figure 3B). On the other hand, the Y654E plasmid mimicked AGEs‐evoked AJ disruption (Figure 3B). These results indicated that β‐catenin Y654 phosphorylation is involved in AGEs‐mediated AJ disruption. To further explore the role of β‐catenin Y654 phosphorylation in AGEs‐mediated endothelial alterations, we investigated whether Y654F plasmids could correct AGEs‐induced hyperpermeability. Indeed, cells pre‐transfected with the Y654F plasmid before stimulation with AGEs exhibited ameliorated hyperpermeability compared with the AGEs group, as indicated by increased TER (Figure 3C) and a decreased permeability coefficient for dextran (Pa) (Figure 3D). Meanwhile, the Y654E plasmid‐pretreated group showed a level of hyperpermeability similar to that of the AGEs group. We, therefore, concluded that β‐catenin Y654 phosphorylation is involved in AGEs‐mediated endothelial monolayer hyperpermeability.

### 3.4 β‐catenin Y142 phosphorylation is responsible for AGEs‐mediated endothelial cytoskeleton remoulding and increased permeability

**FIGURE 4:**
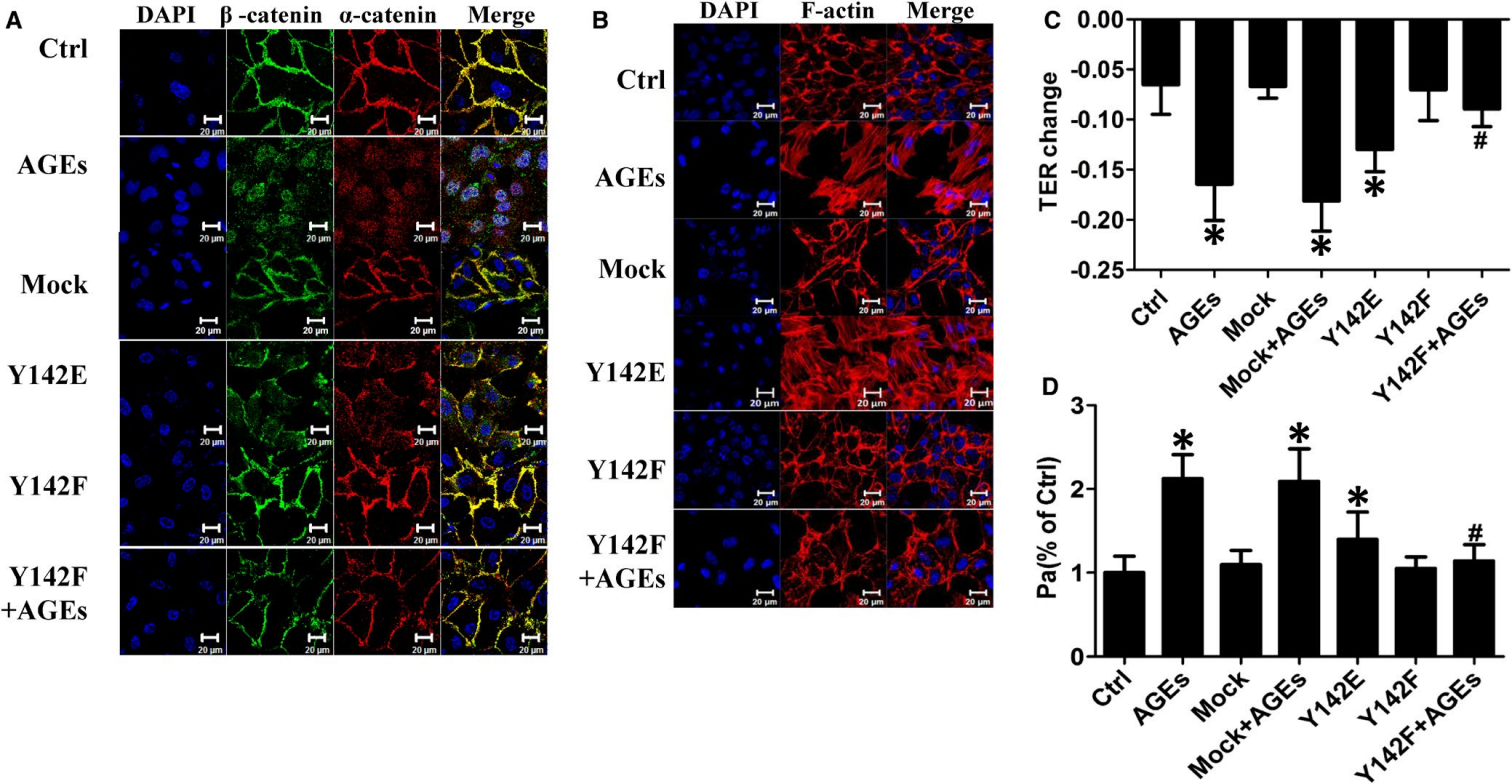
β‐catenin Y142 phosphorylation is required for AGEs‐mediated β‐catenin/α‐catenin dissociation and stress fibre formation and endothelial hyperpermeability. A, B, HUVECs were transfected with indicated overexpression vectors of β‐catenin. Representative confocal images of β‐catenin/α‐catenin and F‐actin were presented after AGEs treatment. C, D, Indicated overexpression vectors were transfected into HUVECs before AGEs stimulation, and TER value and permeability coefficient for dextran (Pa) were detected (n = 5). Data are shown as mean ± SD. *P <.05 versus control, *^#^P* <.05 versus AGEs

For further exploration of the effect of β‐catenin Y142 phosphorylation, β‐catenin Y142 phospho‐deficient (Y142F) and β‐catenin Y142 phospho‐mimic (Y142E) vectors were constructed. HUVECs were pre‐transfected separately with the overexpression vectors followed by 100 μg/mL AGEs stimulation for 8 hours. The immunofluorescence results showed that the Y142F vector ameliorated the disruption of β‐catenin/α‐catenin caused by AGEs, whereas the Y142E plasmid simulated the effect of AGEs (Figure 4A). As previously discussed, F‐actin associates with β‐catenin/α‐catenin complexes; therefore, we next explored whether β‐catenin Y142 phosphorylation influences F‐actin. Indeed, the formation of stress fibres was observed in HUVECs incubated with either AGEs or the Y142E plasmid, whereas this phenomenon was abolished by the Y142F plasmid (Figure 4B). Thus, we concluded that the phosphorylation of β‐catenin Y142 results in β‐catenin dissociation from α‐catenin, cytoskeleton remoulding and the subsequent formation of stress fibres. Based on the vital role of β‐catenin Y142 phosphorylation in this alteration of endothelial structure, we investigated whether it is also involved in AGEs‐induced hyperpermeability. The results for the TER and permeability coefficient for dextran (Pa) indicated that Y142F overexpression attenuated AGEs‐induced endothelial barrier dysfunction, whereas Y142E had a similar effect to that of AGEs (Figure 4C, D). These results indicated that β‐catenin Y142 phosphorylation is another important factor in AGEs‐mediated endothelial hyperpermeability.

### 3.5 β‐catenin Y142 phosphorylation is required for AGEs‐mediated β‐catenin nuclear translocation and ADAM10 expression

**FIGURE 5:**
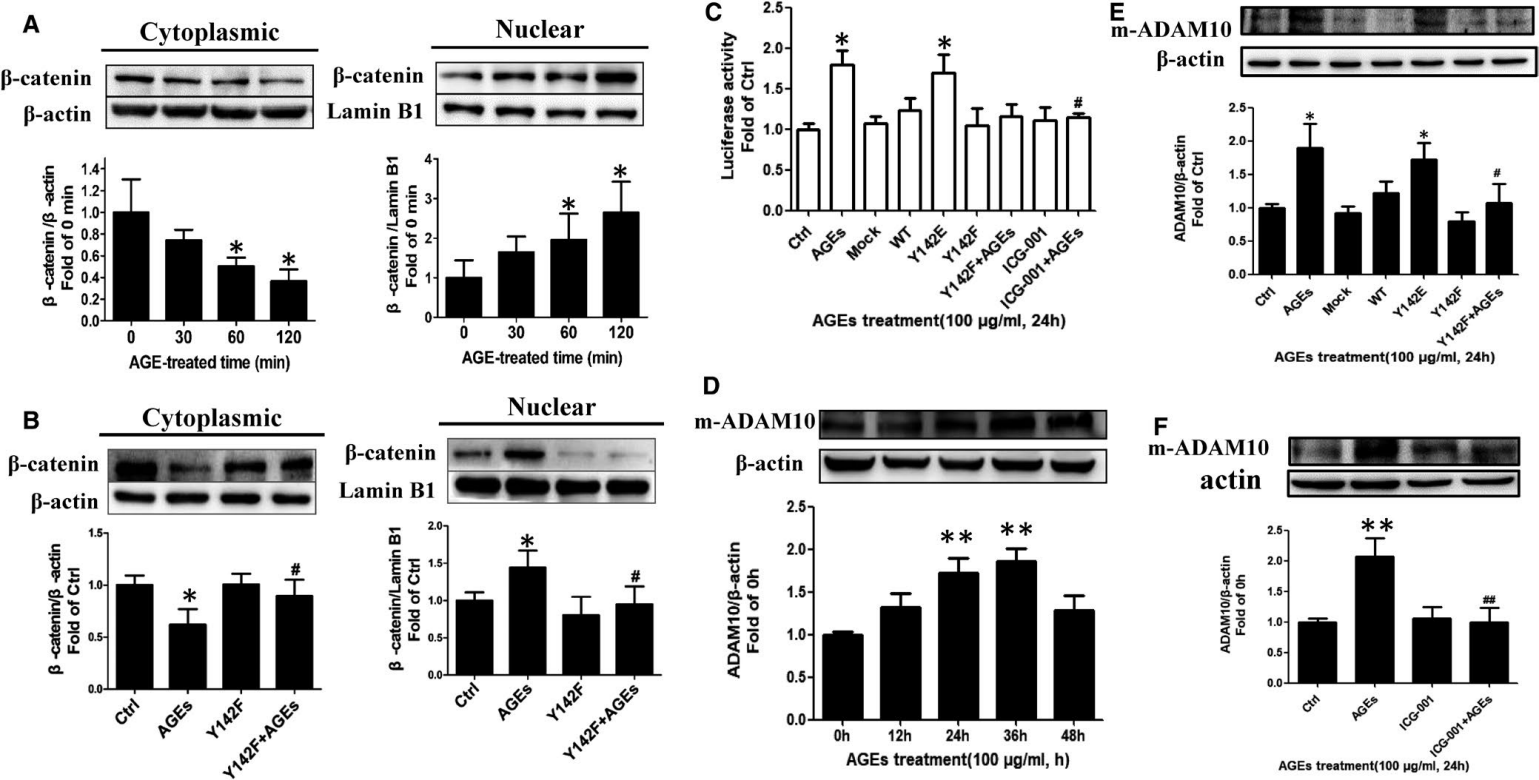
β‐catenin Y142 phosphorylation is required for AGEs‐mediated β‐catenin nuclear translocation and expression of ADAM10. A, AGEs induce β‐catenin nuclear translocation. HUVECs were stimulated by 100 μg/mL AGEs in time‐dependent manner. Cytoplasmic β‐catenin and nuclear β‐catenin were detected by Western blotting (n = 7). Data are shown as mean ± SD. *P <.05 versus 0 min. B, β‐catenin Y142 phosphorylation is required for its nuclear translocation induced by AGEs. HUVECs were transfected with β‐catenin Y142F overexpression vectors followed by AGEs stimulation, cytoplasmic and nuclear β‐catenin were measured (n = 5). Data are shown as mean ± SD. *P <.05 versus control, *^#^P* <.05 versus AGEs. C, AGEs activates TCF/LEF pathway via β‐catenin nuclear translocation and β‐catenin Y142 phosphorylation is required for activation of TCF/LEF pathway induced by AGEs. ICG‐001 is a selective low‐molecular‐weight inhibitor that antagonizes β‐catenin/TCF‐mediated transcription. HUVECs were transfected with indicated overexpression vectors or pretreated with ICG‐001 followed by AGEs stimulation for 24 h. TCF/LEF promoter activity was measured by Dual‐Luciferase Reporter Assay. Luminescence was normalized to Renilla and expressed as fold increase compared with that of control cells (n = 5). Data are shown as mean ± SD. * P <.05 versus ctrl, *^#^ P* <.01 versus AGEs. D, AGEs increase the level of ADAM10 in value. HUVECs were stimulated by 100 μg/mL AGEs in time‐dependent manner, and ADAM10 was detected by Western blotting (n = 7). m‐ADAM10, mature form of ADAM10. Data are shown as mean ± SD. **P <.01 versus 0 h. E, F, β‐catenin Y142 phosphorylation accounts for the increase of ADAM10. Transfected with indicated overexpression vectors or treated with ICG‐001 (10 μmol/L) for 24 h followed by AGEs stimulation for 24h, HUVECs were performed with Western blotting to analyse ADAM10 (n = 5). Data are shown as mean ± SD. * P < .05 versus ctrl, ** *P *< .01 versus Ctrl, ^#^ *P <* .05 versus AGEs

Our previous experiments in this study showed that β‐catenin Y142 phosphorylation results in the dysfunction of its adhesive capacity (Figure 4A, B). Therefore, we next focused on whether it also affected the signal transduction role of β‐catenin. The results showed that with increasing AGEs stimulation time, the cytoplasmic level of β‐catenin decreased and that in the nucleus increased (Figure 5A). This indicated that AGEs mediated the translocation of β‐catenin into the nucleus in a time‐dependent manner. HUVECs with overexpression of the phospho‐deficient β‐catenin Y142 mutant (Y142F) that were then stimulated with AGEs gained increased cytoplasmic β‐catenin and reduced nuclear β‐catenin compared with the AGEs group (Figure 5B). This result indicated that AGEs induced β‐catenin translocation into the nucleus via β‐catenin Y142 phosphorylation. Next, we explored whether β‐catenin in the nucleus activates the TCF/LEF pathway. A dual‐luciferase reporter gene assay indicated that the luciferase activity increased after AGEs stimulation, whereas overexpression of the β‐catenin Y142F plasmid or inhibition of the association between β‐catenin/TCF by ICG‐001 blocked the effect of AGEs (Figure 5C). These data indicated that AGEs activate the TCF/LEF pathway via nuclear translocation of β‐catenin and, thus, promote the expression of Wnt target genes. Next, we checked the expression of other Wnt targets pertaining endothelial permeability, including MMP2, MMP7, MMP9, CCND1 and VEGFA (Figure S3). The results showed that the mRNA level of ADAM10 elevated significantly after AGEs treatment. However, there was no significant change among the other Wnt targets compared with the control group, and thus, we chose ADAM10 for further investigation. Western blotting showed that AGEs up‐regulated the protein levels of ADAM10 in a time‐dependent manner, with a statistically significant difference (*P* <.01) occurring at 24 hours and a return to normal levels at 48 hours (Figure 5D). The β‐catenin Y142F overexpression vector and the ICG‐001 inhibitor were both able to reverse this AGEs‐induced up‐regulation of ADAM10 (Figure 5E, F). These data indicated that β‐catenin Y142 phosphorylation is required for AGEs‐mediated β‐catenin nuclear translocation and targets to ADAM10.

### 3.6 β‐catenin Y142 phosphorylation involvement in AGEs‐stimulated VE‐cadherin shedding by ADAM10

**FIGURE 6:**
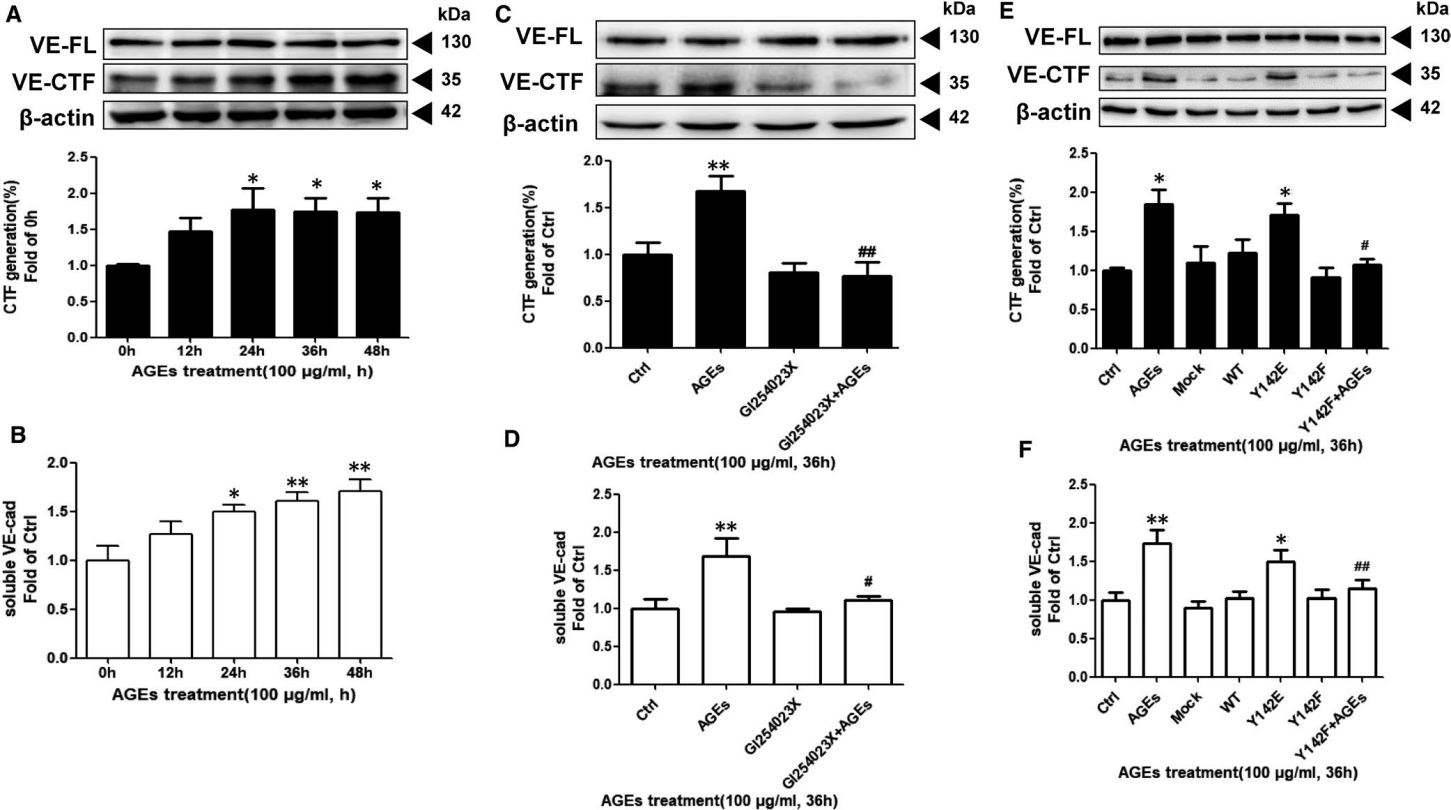
β‐catenin Y142 phosphorylation involvement in AGEs stimulated VE‐cadherin shedding by ADAM10. A, B, HUVECs were stimulated by 100 μg/mL AGEs in time‐dependent manner. The generation of VE‐cadherin CTF was measured by Western blotting using C‐terminal VE‐cadherin antibody and the release of soluble VE‐cadherin ectodomain was analysed by ELISA according to the instruction of the manufacturer. (n = 5). VE‐FL, full length of VE‐cadherin; VE‐CTF, C‐terminal fragment of VE‐cadherin. Data are shown as mean ± SD. * P <.05 versus 0h. ** *P* <.01 versus 0 h. C, D, HUVECs were stimulated by 100 μg/mL AGEs for 36h in the presence of ADAM10 inhibitor GI254023X (10 μmol/L) and γ‐secretase inhibitor DAPT (10 μmol/L ). Similarly, immunoblotting measured the generation of VE‐cadherin CTF and ELISA measured the release of soluble VE‐cadherin ectodomain (n = 5). Data are shown as mean ± SD. ** *P* < .01 versus 0 h. E, F, HUVECs were transfected with indicated overexpression vectors. After AGEs stimulation for 36h, immunoblotting measured the generation of VE‐cadherin CTF and ELISA measured the release of soluble VE‐cadherin ectodomain (n = 4). Data are shown as mean ± SD. ** *P <* .01 versus Ctrl, ^##^ *P <* .01 versus AGEs

Having shown that β‐catenin Y142 phosphorylation is involved in AGEs‐induced nuclear translocation of β‐catenin and increased ADAM10 expression, we next investigated whether AGEs lead to VE‐cadherin shedding by ADAM10 and what role β‐catenin Y142 phosphorylation plays in this process. The generation of the VE‐cadherin C‐terminal fragment (CTF) in the membrane and that of the soluble VE‐cadherin ectodomain in the cellular supernatant were measured by western blotting and ELISA, respectively. As VE‐cadherin CTF is a substrate for subsequent γ‐secretase cleavage, the HUVECs used in our assays were treated in the presence of the γ‐secretase inhibitor, DAPT (10 μmol/L).^27^ We observed a time‐dependent increase in the production of VE‐cadherin CTF following AGEs treatment that was concomitant with elevated soluble VE‐cadherin (Figure 6A, B), indicating that AGEs mediate VE‐cadherin cleavage. Next, HUVECs were treated with AGEs in the presence or absence of a selective ADAM10 inhibitor, GI254023X. Both the production of VE‐cadherin CTF and the release of soluble VE‐cadherin caused by AGEs were diminished in the presence of this inhibitor (Figure 6C, D). Consistent with the chemical inhibitor results, the increased production of VE‐cadherin CTF caused by AGEs were diminished after knock‐down of ADAM10 (Figure S4A,B). Taken together, it is suggested that ADAM10 is involved in AGEs‐stimulated VE‐cadherin shedding. Finally, we overexpressed the β‐catenin Y142F phospho‐deficient mutant followed by AGEs incubation for 36 hours, and the results showed that Y142 abolished the AGEs‐induced increase in both VE‐cadherin CTF and soluble VE‐cadherin compared with the AGEs group (Figure 6E, F). Thus, it is proved that the involvement of β‐catenin Y142 phosphorylation in AGEs‐stimulated VE‐cadherin shedding occurs via its targeting of ADAM10.

### 3.7 Tyrosine phosphorylation of β‐catenin at either the Y654 or Y142 residue is required for AGEs‐induced microvascular hyperpermeability *in vivo*

**FIGURE 7:**
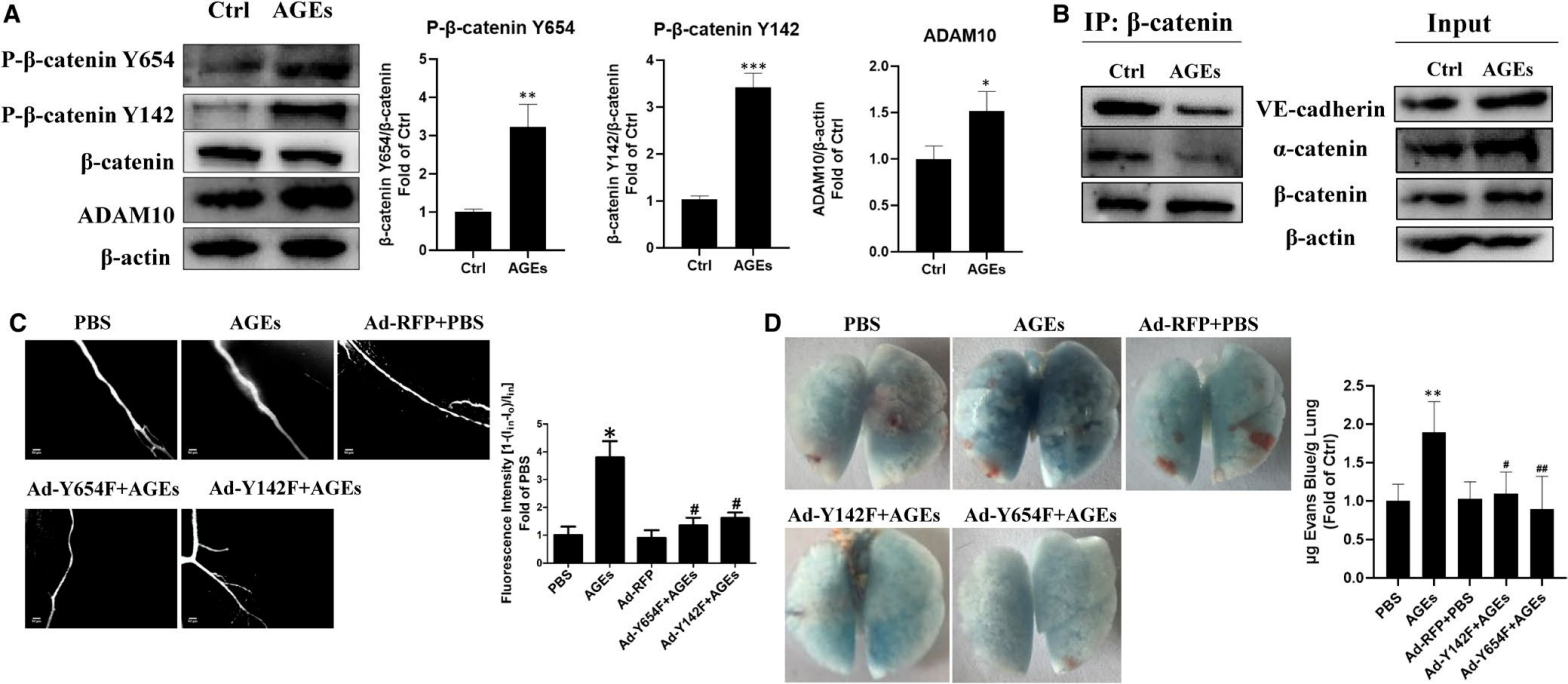
Involvement of β‐catenin Y654 and Y142 phosphorylation in AGEs‐mediated microvascular hyperpermeability in vivo. A, C57 mice were administrated AGEs (10 mg/kg) via i.p. for 7 consecutive days, and then mesenteric vessels were separated to test the protein level of β‐catenin Y654, Y142 phosphorylation and ADAM10 by WB (n = 4). Data are shown as mean ± SD. **P < .01 versus control, ***P < .001 versus control, B, Co‐immunoprecipitation assay was performed to observe the colocation of VE‐cadherin, α‐catenin and β‐catenin of the mesenteric vessels, with the results shown in the IP column, whereas total proteins were shown in the Input column. C, Ad‐Y654F and Ad‐Y142F were intravenously administered to C57 mice that were intraperitoneally injected with AGEs (10 mg/kg) after 2 days for 7 consecutive days. Mesentery vascular permeability is expressed as the relative ﬂuorescent intensity inside the vessel to that outside the vessel (n = 4). Data are shown as mean ± SD. *P < .05 versus control, *^#^P* < .05 versus AGEs. Five representative images were shown at a 100× magnification. (D) Lung vascular permeability is expressed as Evans blue (EB) concentration inside the lung tissue two hours after intravascularly injected EB (n = 4). Five representative images were shown, and quantity data are shown as mean ± SD. *P < .05 versus control, *#P* < .05 versus AGEs, *##P* < .01 versus AGEs

To further confirm the protective role of Y654 phospho‐deficient β‐catenin in microvascular permeability, an overexpression adenovirus vector (Ad‐Y654F) was constructed and administered to C57 mice followed by the intraperitoneal injection of AGEs (10 mg/kg) for seven consecutive days. The transfection efficiency of indicated adenovirus in mesenteric vessels and lung was verified and shown in Figure S5B,C. The dextran flux across the mesenteric microvessels was then monitored. The results showed that, compared with the control (PBS‐injected mice), the AGEs‐treated mice presented higher dextran leakage, whereas Ad‐Y654F drastically reversed AGEs‐evoked dextran extravasations (Figure 7C). C57 mice injected with Ad‐Y142F showed similar results (Figure 7C). Meanwhile, by measuring the extravasation of Evans Blue(EB) into the interstitial tissue of lung, we observed increased EB extravasation in the lungs in response to AGEs stimulation, indicating that AGEs resulted in pulmonary vascular barrier dysfunction (Figure 7D). Mice overexpressing Y142F or Y654F prior to stimulation of AGEs had decreased EB extravasation through alveolar microvessels (Figure 7D). Taken together, these data point towards the key role of β‐catenin Y654 and Y142 phosphorylation in AGEs‐induced endothelial hyperpermeability.

Moreover, the two tyrosine residues phosphorylation level of β‐catenin Y654 and Y142 site in mesenteric vein are both increased compared with the control group(Figure 5A). At the same time, CO‐IP assay results showed that the decreased co‐localization of VE‐cad/β‐catenin/α‐catenin complexes after AGEs stimulation (Figure 5B). AGEs also induced elevated ADAM10 expression in mesenteric vein (Figure 5A). The results above were consistent with the ones in vitro, which strengthen the in vitro findings in this study.

## 4 DISCUSSION

Consistent with previous reports, the present study showed that AGEs are involved in endothelial hyperpermeability. However, the precise mechanism remained to be uncovered. It has been widely accepted that the precise regulation of endothelial permeability depends on the balance between intercellular adhesion forces and intracellular contraction forces.^8^, ^9^ Either the impairment of adhesion forces caused by the disruption of AJs or enhancement of the centralized contraction forces caused by stress fibre formation can contribute towards endothelial hyperpermeability.

Based on our observations of the key role of AGEs in the dissociation of VE‐cadherin/β‐catenin and the impairment of AJs, which is consistent with the work by Otero et al,^6^ we chose to investigate the role of β‐catenin in AGEs‐induced dysregulation of the AJs. Recent studies have shown a connection between the phosphorylation of β‐catenin and cadherin functional regulation,^16^, ^33^ and these two molecules mainly comprise the AJs.^34^ At least three critical phosphorylated tyrosine residues promoting β‐catenin signalling have been well studied; among these, Y86 plays a role in stabilizing β‐catenin, Y333 promoting nuclear function in response to EGF, Y142 reducing α‐catenin/β‐catenin binding, whereas Y654 is considered essential for the disruption of E‐cadherin/β‐catenin.^17^, ^19^, ^33^, ^35^ This effect was not only shown in several cell lines^35^, ^36^, ^37^ but also confirmed in the in vivo study performed by van Veelen *et al* using a conditional knock‐in mouse model expressing a phospho‐mimic Y654 β‐catenin mutant.^18^ More in‐depth molecular analysis showed that β‐catenin shields the PEST sequence on E‐cadherin, preventing it from ubiquitination and degradation.^17^, ^33^, ^38^ When Y654 is phosphorylated, it generates a negative charge that clashes with the nearby aspartate residues of cadherin and, hence, collapses the cadherin‐β‐catenin interaction.^14^, ^15^, ^17^

Here, focusing on AJ association and considering that the β‐catenin Y654 and Y142 residues phosphorylation reduces β‐catenin/VE‐cadherin and β‐catenin/α‐catenin binding, respectively, whereas other residues including Y86 and Y333 do not reported such effect, we chose these two tyrosine residues for this research study. Firstly, we showed that the enhanced β‐catenin Y654 phosphorylation in HUVECs induced by AGEs treatment led to AJ disruption and subsequent increases in endothelial permeability. A β‐catenin Y654 phospho‐deficient mutant plasmid (Y654F) abrogated this effect of AGEs on the AJs. Conversely, a β‐catenin Y654 phospho‐mimic plasmid (Y654E) triggered the dissociation of VE‐cadherin/β‐catenin in HUVECs. These data suggested that β‐catenin Y654 phosphorylation is a prerequisite for the AGEs‐evoked collapse of the AJs. Consistent with this, we found that Y654F prevented AGEs‐evoked endothelial monolayer hyperpermeability and rescued mesenteric microvascular hyperpermeability. These data point towards the key role of β‐catenin Y654 phosphorylation in AGEs‐induced endothelial dysfunction.

However, the molecular mechanism underlying AGEs‐induced AJ collapse still needed to be elucidated. As an important member of the largest non‐receptor tyrosine kinase family, Src is known to be activated by AGEs and to participate in endothelial hyperpermeability.^12^ In epithelial cells, Src is required for β‐catenin Y654 phosphorylation.^39^ A recent study further showed that Src activity is responsible for β‐catenin activity and release from E‐cadherin.^40^ Considering the vital role of Src in signalling transduction and the possibility of Src acting as an upstream effector of β‐catenin phosphorylation, we next investigated whether Src is responsible for AGEs‐induced β‐catenin Y654 phosphorylation. Indeed, our results showed that both pharmacological inhibition and RNA silencing of Src prevented AGEs‐induced β‐catenin Y654 phosphorylation. These data confirmed that the activation of Src is essential for AGEs‐induced β‐catenin Y654 phosphorylation.

Next, we shifted our focus to Y142 of β‐catenin, another phosphorylated residue. In the AJs, α‐catenin tends to form a complex with β‐catenin rather than with itself.^41^ Homodimers of α‐catenin can compete with Arp2/3, an actin‐binding protein, leading to the regulation of cytoskeleton remoulding.^42^ This outcome is accomplished parallel to the interaction of α‐catenin with α‐actin and vinculin.^43^, ^44^ Previous research has shown that amino acid mutants at Y142 of β‐catenin can block its association with α‐catenin.^43^, ^44^ Thus, we aimed to uncover the effect of Y142 phosphorylation on its contribution towards the remoulding of F‐actin in response to AGEs. In the second part of the present study, the introduction of a β‐catenin Y142‐phospho‐deficient mutant plasmid reversed the dissociation of β‐catenin from α‐catenin as well as the contraction of F‐actin caused by AGEs, thereby improving monolayer permeability. Conversely, a β‐catenin Y142‐phospho‐mimic plasmid led to the dissociation of β‐catenin and α‐catenin, the formation of stress fibres, and increased monolayer permeability. Together with the result that β‐catenin Y142F rescued mesenteric microvascular hyperpermeability, we concluded that Y142 phosphorylation is responsible for the reorganization of cytoskeletal filaments and increased permeability induced by AGEs.

FAK is a focal adhesion‐associated protein kinase and has been widely investigated in terms of its modulation of endothelial permeability. FAK directly phosphorylates β‐catenin at Y142 and mediates VEGF‐induced hyperpermeability.^45^ Previous research by our group demonstrated that FAK can be activated by the AGEs‐RAGE‐Src pathway^12^ ; here, we found that a pharmacological inhibitor of FAK blocked AGEs‐induced β‐catenin phosphorylation at Y142. In summary, AGEs may exert its pathophysiological effect in part through FAK.

It is widely accepted that β‐catenin is not only a structural component of cadherin‐based adherens junctions but that it also acts as a key nuclear effector of canonical Wnt signalling in the nucleus.^17^ Upon destruction of the AJ structure, β‐catenin is released. This free β‐catenin in the cytoplasm is recognized by the Axin complex, which includes the scaffold protein Axin, protein phosphatase 2A, adenomatous polyposis coli, β‐TrCP, CK1 and glycogen synthase kinase 3 β (GSK3B).^46^ β‐catenin is phosphorylated by CK1 and GSK3B to mediate its rapid degradation, and this process can be reversed by extracellular Wnt signalling.^17^ β‐catenin can be transferred into the nucleus with the help of the B‐cell CLL/lymphoma 9 (BCL9) protein where it binds to the transcription factors, TCF‐LEF, to initiate the expression of Wnt‐related genes.^25^ In addition, it has been reported that β‐catenin can mediate the expression of VEGF^47^ and DII4,^48^ and the high expression of VEGF can also be detected in endothelial cells stimulated by AGEs.^49^ Some studies have indicated that β‐catenin Y142 phosphorylation can induce β‐catenin translocation into the nucleus, thus promoting the expression of downstream genes.^16^ Consistent with this, we observed AGEs‐mediated β‐catenin nuclear translocation and TCF/LEF pathway activation, along with decreased β‐catenin levels in the cytoplasm and concomitantly increased levels in the nucleus. The results also showed that β‐catenin Y142 phosphorylation is required for this process. However, despite intensive investigation, the detailed underlying mechanisms regulating β‐catenin nuclear transport still need to be further investigated.

The integrity of adherens junctions can also be affected by several proteases via the cleavage of the extracellular or intracellular domains of cadherins.^50^ As a surface‐expressed protease, ADAM10 mediates the cleavage of vascular surface molecules at an extracellular site close to the membrane.^51^ ADAM10‐mediated cleavage of E‐ and N‐cadherin in keratinocytes or neuronal cells causes cells to become motile and invasive.^51^ Further works have also identified ADAM10 as a regulator of vascular permeability by direct proteolytic cleavage of VE‐cadherin at its ectodomain site, releasing a soluble fragment and generating a carboxyl‐terminal membrane‐bound stub.^7^, ^27^ In the present study, we found endothelial permeability to be associated with ADAM10‐mediated cleavage of VE‐cadherin: AGEs trigger the up‐regulation of ADAM10, which increases the generation of VE‐cadherin CTFs and soluble VE‐cadherin. Interestingly, there was no significant mRNA level change among the other Wnt targets including MMP2, MMP7, MMP9, CCND1 and VEGFA, suggesting that these genes may not be involved in AGEs‐induced hyperpermeability. However, we did not detect the protein level of these genes, which may regulate endothelial permeability by post‐transcriptional mechanism that needs further investigation. On the other hand, β‐catenin Y142 phosphorylation plays a critical intracellular role in the up‐regulation of ADAM10. Our study demonstrates that the involvement of β‐catenin Y142 phosphorylation in AGEs‐mediated β‐catenin nuclear translocation is exerted via its targeting of ADAM10, which stimulates VE‐cadherin shedding. In other words, AGEs can directly mediate AJ structure changes, and in addition, the up‐regulation of ADAM10 can be mediated by β‐catenin signal transduction, thus further weakening the AJ structure. Of note, it is unknown whether ADAM10‐induced VE‐cadherin ectodomain cleavage causes its cytoplasmic disassociation from β‐catenin, which, in turn, leads to increased free β‐catenin in the cytoplasm that may amplify β‐catenin signalling. This is a possible positive feedback mechanism that needs to be further investigated.

**FIGURE 8:**
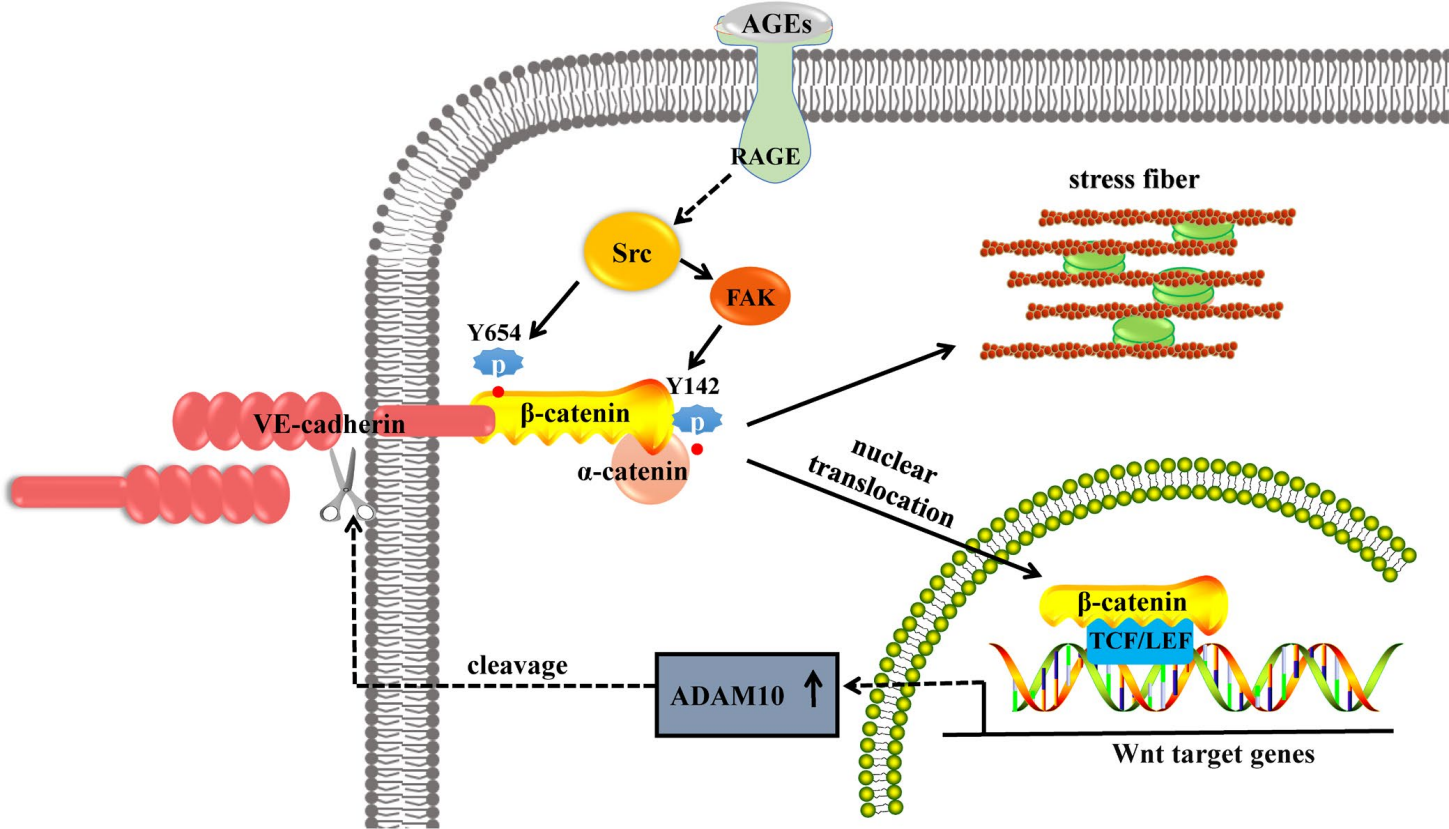
Schematic representation describing the role of β‐catenin in AGEs‐induced endothelial hyperpermeability. AGEs, binding with RAGE, mediate Src activation that, in turn phosphorylates β‐catenin at Y654. This results in dissociation of VE‐cadherin/β‐catenin, impairing cell‐cell adhesion. AGEs facilitate FAK phosphorylation that, in turn, phosphorylates β‐catenin at Y142, causing disruption of β‐catenin/α‐catenin and contributing to cytoskeleton reorganization, with cortical actin relocating into stress fibres. Furthermore, phosphorylates β‐catenin at Y142 leads to β‐catenin accumulation and nuclear translocation. The nuclear‐located β‐catenin combines to TCF/LEF and contributes to the transcription of Wnt target genes ADAM10, which mediates VE‐cadherin shedding and leads to further impairment of AJs. The weakened adhesion forces resulting from impaired AJs, complemented by strengthened contraction forces derived from stress fibre formation, lead to endothelial hyperpermeability

In summary, in the current study, we focused on the effect of β‐catenin phosphorylation at Y654 and Y142 on AGEs‐induced endothelial hyperpermeability. We demonstrated the following: (i) Y654 phosphorylation as triggered by Src is responsible for the destabilization of endothelial AJs; (ii) Y142 phosphorylation as stimulated by FAK activation leads to the reorganization of F‐actin, endothelial contraction, β‐catenin nuclear translocation, and up‐regulated transcription of the Wnt target, ADAM10, which cleaves VE‐cadherin. These cellular processes act in concert to contribute towards endothelial barrier dysfunction in response to AGEs (Figure 8). Overall, β‐catenin is phosphorylated and then activated in the presence of AGEs, resulting in the dissociation of adhesion junction among VE‐cadherin/β‐catenin/α‐catenin and up‐regulation of ADAM10. These cause hyperpermeability of endothelial cells ultimately. Therefore, our study provides further mechanistic insights into the cellular maladaptation involved in diabetic microvascular complications.

## CONFLICT OF INTEREST

No conflicts of interest, financial or otherwise, are declared by all authors.

## AUTHOR CONTRIBUTION

**Jie Weng:** Investigation (equal); Methodology (equal); Project administration (equal); Visualization (equal); Writing‐original draft (supporting). **Zhenfeng Chen:** Investigation (equal); Methodology (equal); Project administration (equal); Visualization (equal); Writing‐original draft (supporting). **Jieyu Li:** Investigation (equal); Methodology (equal); Project administration (equal); Visualization (equal). **Qi He:** Methodology (supporting); Validation (supporting); Visualization (supporting); Writing‐review & editing (supporting). **DeShu Chen:** Writing‐original draft (lead). **Lin Yang:** Conceptualization (supporting); Formal analysis (supporting); Investigation (supporting). **Haiying Su:** Project administration (supporting). **Junlin Huang:** Project administration (supporting). **Shengxiang Yu:** Project administration (supporting). **Qiaobing Huang:** Conceptualization (supporting); Supervision (supporting). **Qiulin Xu:** Conceptualization (lead); Supervision (lead). **Xiaohua Guo:** Conceptualization (lead); Funding acquisition (lead); Supervision (lead); Validation (lead); Writing‐review & editing (lead).

## ETHICAL APPROVAL

The using mice were approved by the Animal Care Committee of the Southern Medical University of China and in strict accordance with the Guide for the Care and Use of Laboratory Animals of the National Institutes of Health.

## Supporting information

Fig S1

Fig S2

Fig S3

Fig S4

Fig S5

## Data Availability

All data used or analysed during this study are included in this published article.
