# Supplementary figures and images for: Advanced glycation end products induce endothelial hyperpermeability via β‐catenin phosphorylation and subsequent up‐regulation of ADAM10
Source: J Cell Mol Med. 2021 Jul 5;25(16):7746–59. doi: 10.1111/jcmm.16659 (PMC8358892; doi:10.1111/jcmm.16659)

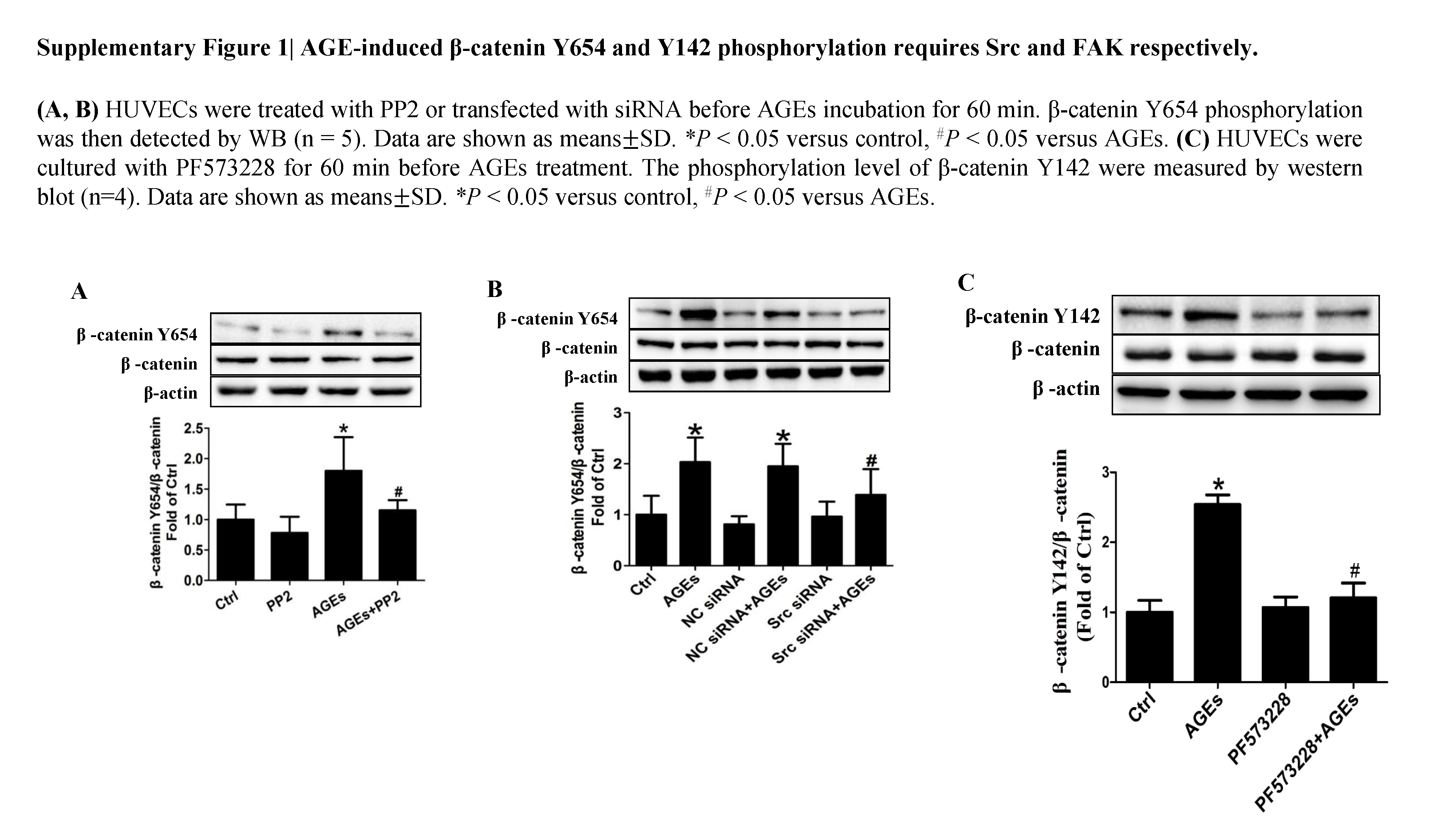

Supplement: Supplementary file 1 — Fig S1 [file JCMM-25-7746-s004.tif]

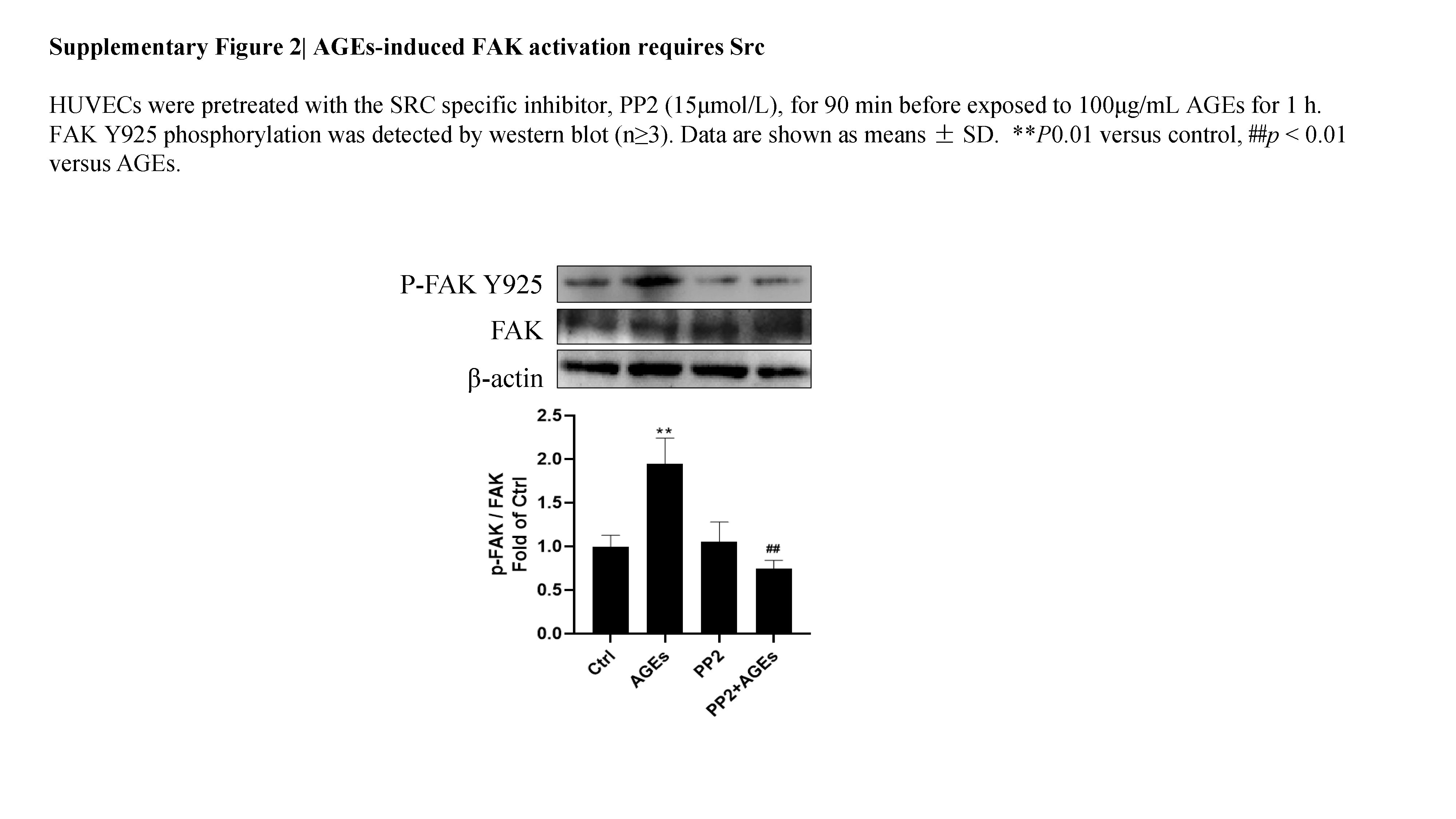

Supplement: Supplementary file 2 — Fig S2 [file JCMM-25-7746-s001.tif]

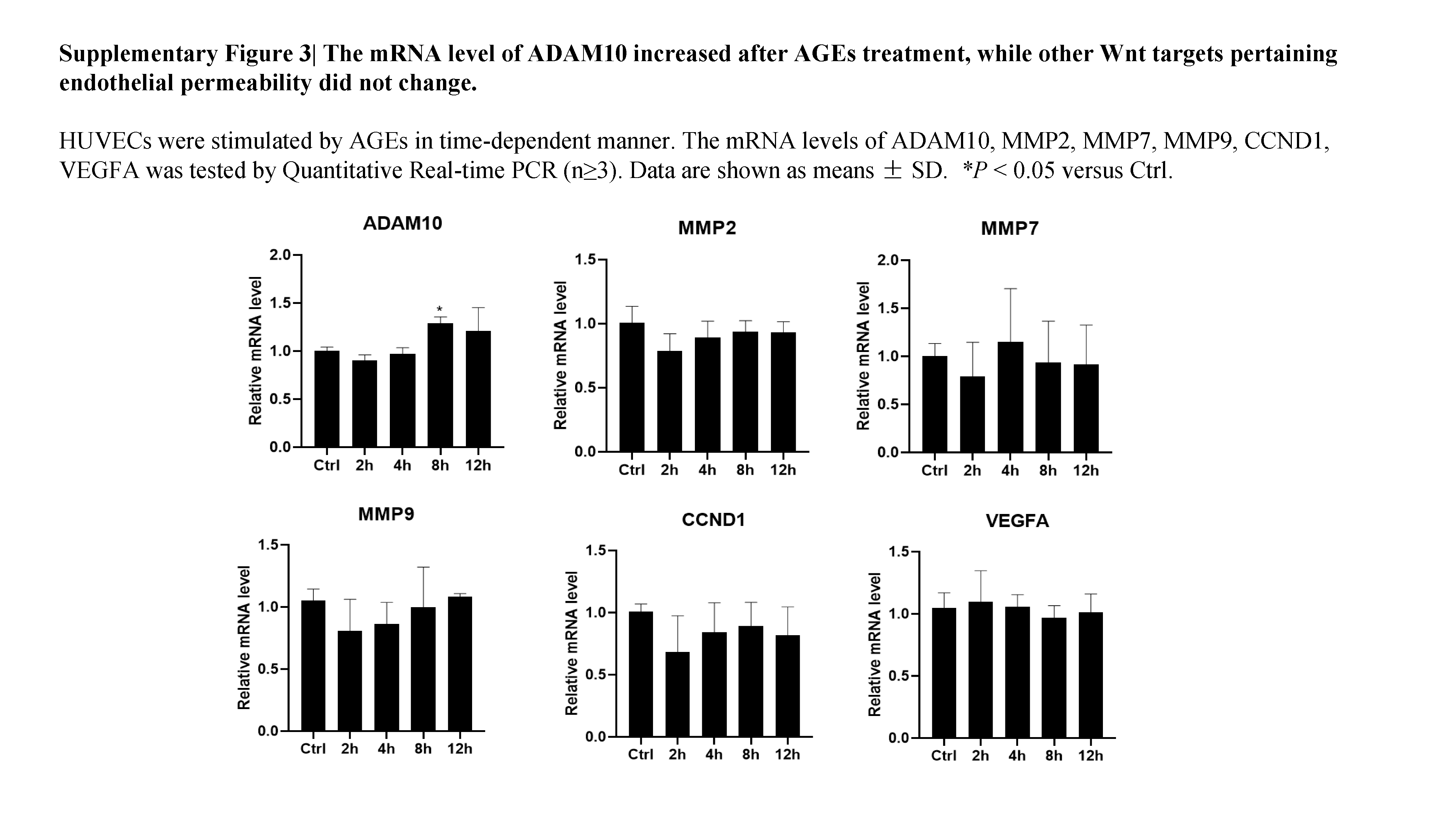

Supplement: Supplementary file 3 — Fig S3 [file JCMM-25-7746-s003.tif]

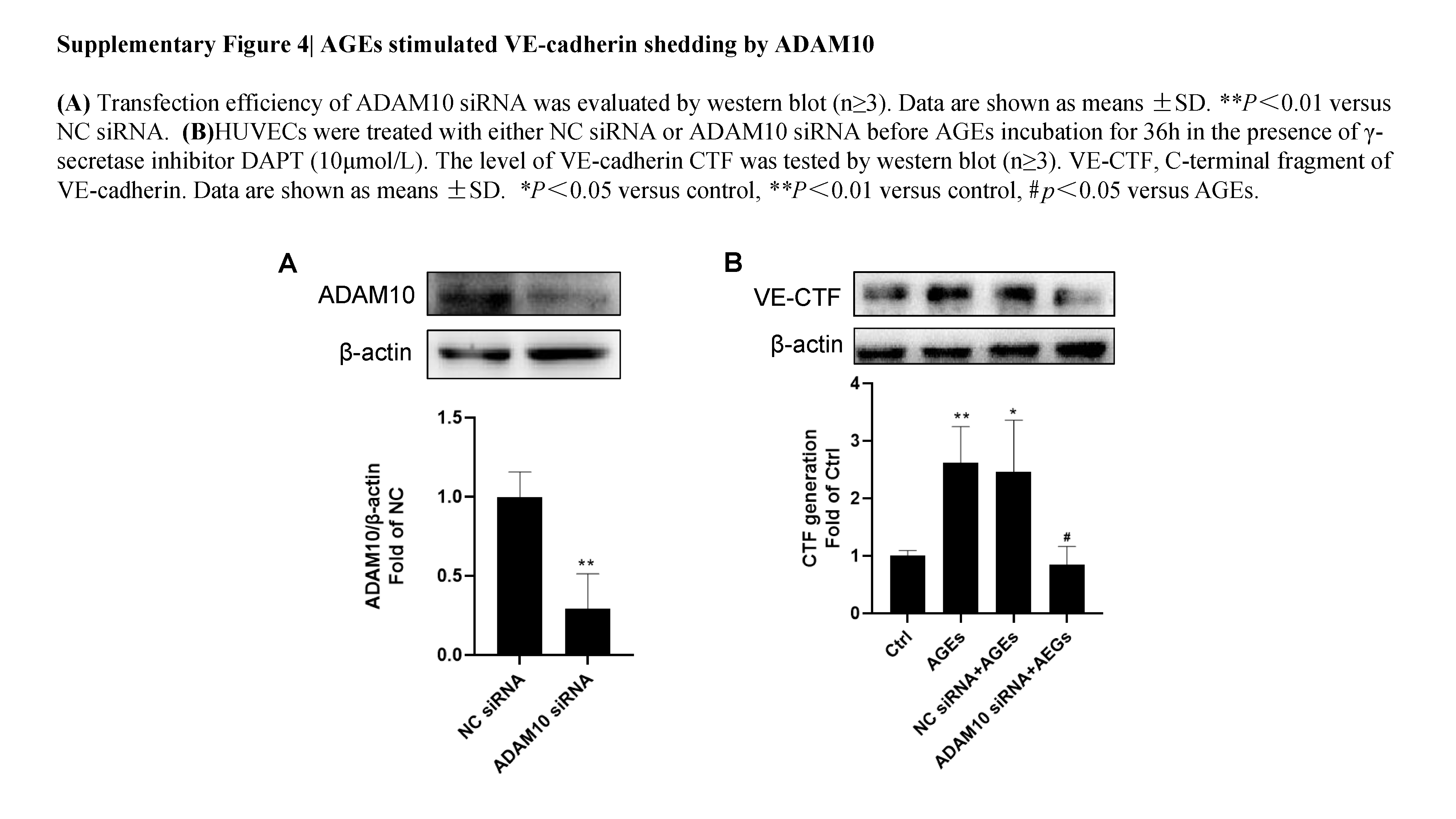

Supplement: Supplementary file 4 — Fig S4 [file JCMM-25-7746-s005.tif]

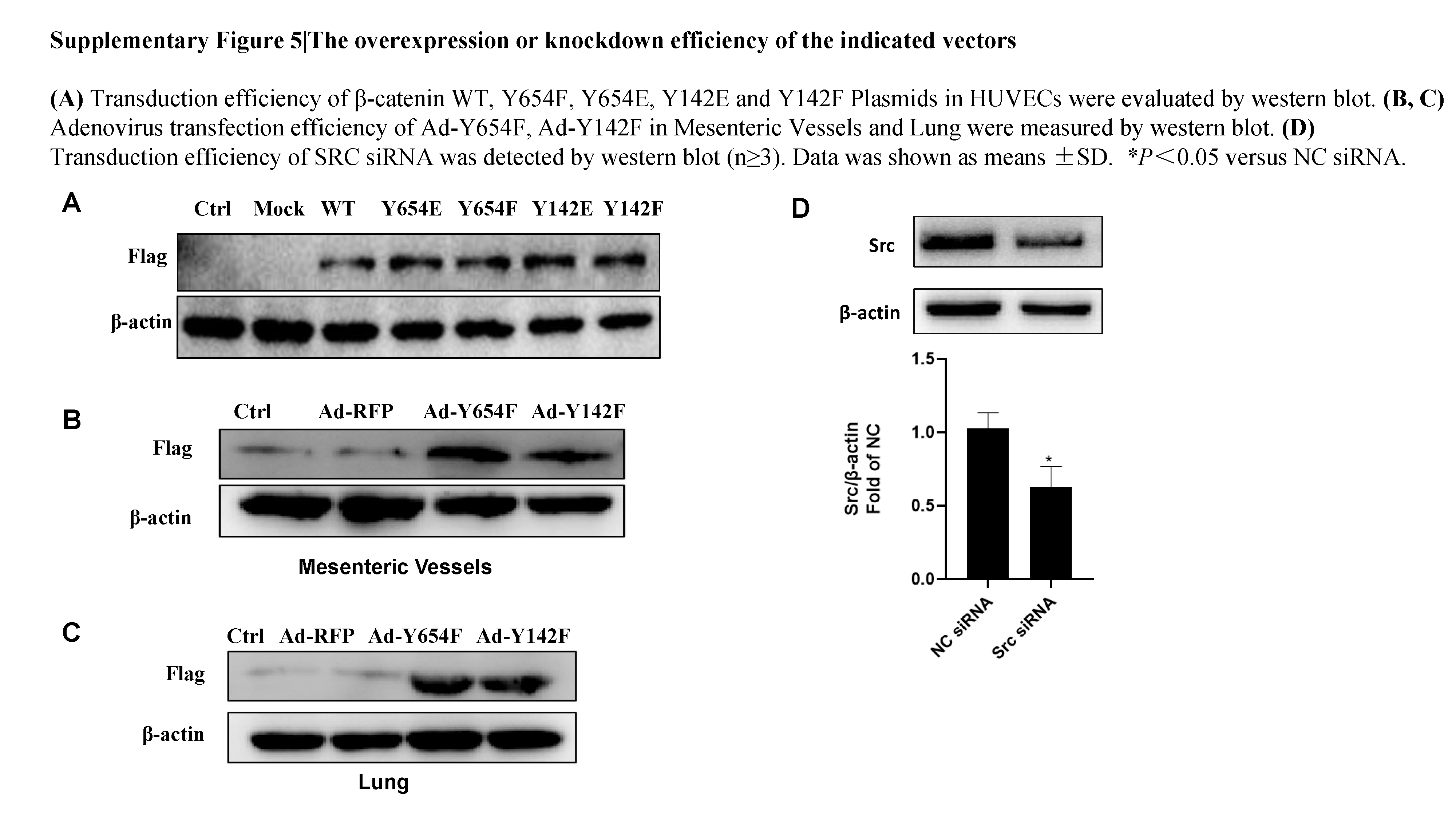

Supplement: Supplementary file 5 — Fig S5 [file JCMM-25-7746-s002.tif]
